# Quality by design-based approach for the development of an analytical method for quantifying ponatinib in rat plasma

**DOI:** 10.1016/j.heliyon.2024.e38637

**Published:** 2024-09-28

**Authors:** Nahyun Koo, Eun Ji Lee, Min Ju Kim, Minjung Park, Kyeong-Ryoon Lee, Yoon-Jee Chae

**Affiliations:** a College of Pharmacy, Woosuk University, Wanju, 55338, Republic of Korea; b Laboratory Animal Resource Center, Korea Research Institute of Bioscience and Biotechnology, Cheongju, 28116, Republic of Korea; c Department of Biotechnology, University of Science and Technology, Daejeon, 34113, Republic of Korea; d Research Institute of Pharmaceutical Sciences, Woosuk University, Wanju, 55338, Republic of Korea

**Keywords:** Ponatinib, Analytical quality by design, Taguchi method, Box-Behnken design, Method development and validation

## Abstract

Ponatinib is a potent tyrosine kinase inhibitor that is approved for the treatment of chronic myeloid leukemia and Philadelphia chromosome-positive acute lymphoblastic leukemia. To further expand its clinical applications, accurate quantification of ponatinib in plasma is essential. In this study, we developed and validated a sensitive and selective high-performance liquid chromatography (HPLC) method coupled with a fluorescence detector (FLD) to measure ponatinib concentrations in rat plasma using the Analytical Quality by Design approach. Briefly, we screened and optimized the critical method parameters using the Taguchi and Box-Behnken designs. The developed method had excellent linearity in the range of 1–1000 ng/mL, sensitivity, and reproducibility, and required minimal sample volume and a short run time. Compared with previously reported HPLC-ultraviolet (UV) and liquid chromatography-tandem mass spectrometry (LC-MS/MS) methods, this HPLC-FLD method offers superior sensitivity, simpler sample preparation, and greater efficiency. We successfully used this method in a pharmacokinetic study in rats to obtain reliable data on ponatinib plasma concentrations. Altogether, this analytical method will be applicable in several analytical conditions and will support further pharmacokinetic and clinical investigations of ponatinib for various cancer treatments.

## Graphical abstract

**Figure:**
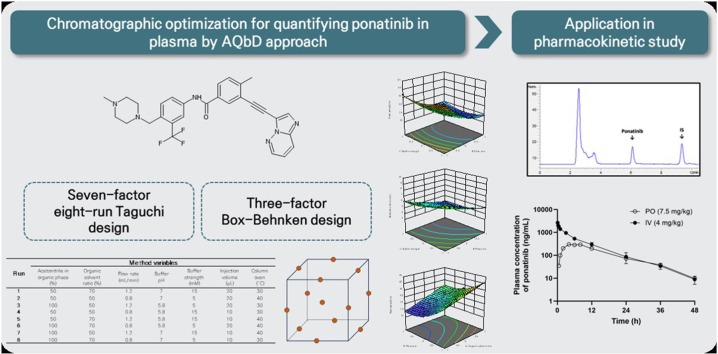

## 1 Introduction

Ponatinib (Iclusig®) is an oral tyrosine kinase inhibitor (TKI) that is approved for use in patients with chronic myeloid leukemia (CML) and Philadelphia chromosome-positive acute lymphoblastic leukemia (Ph + ALL) that are either resistant or intolerant to other TKIs [1]. CML accounts for approximately 15–20 % of all adult patients with leukemia and Ph + ALL is found in approximately 20–30 % of adult ALL cases [2,3]. Given these prevalence, ponatinib plays a critical role in managing patients with these aggressive forms of leukemia, particularly those who develop resistance to other TKIs. Ponatinib effectively inhibits Bcr-Abl, including mutant forms that are resistant to other TKIs, such as the T315I mutation [4]. In the EPIC trial, a multicenter, international, phase 3, randomized, open-label study, treatment with ponatinib led to higher molecular response rates than treatment with imatinib, proving its superior efficacy [5]. Recent studies have attempted to extend the use of ponatinib beyond the treatment of CML [6]. These studies highlighted the broad kinase inhibition capabilities of ponatinib, particularly for targets, such as FGFRs, RET, AKT, ERK1/2, KIT, and MEKK2 [[7], [8], [9]]. Consequently, the efficacy of ponatinib has been evaluated in cancers in which these kinases play key roles, including thyroid, breast, ovarian, lung cancers, neuroblastoma, and rhabdoid tumors [[10], [11], [12]]. Ongoing clinical trials are investigating the efficacy of ponatinib for the treatment of FLT3-ITD acute myelogenous leukemia, head and neck cancers, and gastrointestinal stromal tumors [6] (see Table 5, Table 6).

Analytical Quality by Design (AQbD) is a systematic approach to develop and validate analytical methods in the pharmaceutical industry [[13], [14], [15], [16], [17]]. In contrast to traditional methods, which rely heavily on end-product testing, AQbD emphasizes embedding quality into analytical processes from the beginning, enhancing the robustness, consistency, and regulatory compliance of analytical procedures. By systematically defining the Analytical Target Profile (ATP) and comprehensively understanding the critical factors influencing method performance, AQbD ensures that methods are less susceptible to variations and maintains the integrity of the analytical results across different batches and conditions. Regulatory authorities, such as the Food and Drug Administration (FDA) of the United States and the European Medicines Agency (EMA), encourage the use of AQbD for systematic risk management and reproducibility of analytical methods [18]. In the context of bioanalytical methods, such as pharmacokinetic analysis, the implementation of AQbD is critical. Accurate and reliable pharmacokinetic data are essential for understanding the absorption, distribution, metabolism, and excretion (ADME) properties of drugs, as they directly affect dosing regimens and therapeutic efficacy. The implementation of AQbD in bioanalytical methods requires the use of advanced statistical tools, such as the Design of Experiments (DoE), to optimize method development, resulting in the production of more reliable and reproducible information.

Accurate measurement of ponatinib concentrations in plasma is essential for determining the optimal dose for each cancer type to maximize therapeutic efficacy and minimize adverse effects. Furthermore, accurate information on drug concentrations within the body is critical for extending the therapeutic application of ponatinib to various cancers. Such pharmacokinetic investigations are crucial for delineating safety profiles, aiding in the determination of the therapeutic window and toxicity thresholds across different patient populations, and ultimately enhancing the clinical usability of drugs. Liquid chromatography-tandem mass spectrometry (LC-MS/MS) has been used to analyze ponatinib in plasma [19,20]. Although LC-MS/MS is the gold standard, due to its sensitivity and specificity, the development of various analytical methods is advantageous. The availability of multiple analytical methods enables flexibility and allows laboratories to select the most appropriate method based on specific conditions, resources, and expertise. Furthermore, the use of various validated methods enables cross-validation of results, thereby improving the reliability and robustness of the analytical data. High-performance liquid chromatography (HPLC) coupled with a fluorescence detector (FLD) is a cost-effective and relatively simple-to-operate method for analyzing fluorescent compounds; thus, this approach is particularly attractive for routine analysis in various settings.

The aim of this study was to develop a method to analyze ponatinib in rat plasma using HPLC-FLD following the guidelines of the FDA and EMA [21,22]. By applying the AQbD approach, we systematically developed a method with improved robustness. This approach enables a comprehensive understanding of the factors influencing method performance, ensuring consistent and reliable analytical results across different laboratories.

## 2 Materials and method

### 2.1 Materials and method

**Fig. 1:**
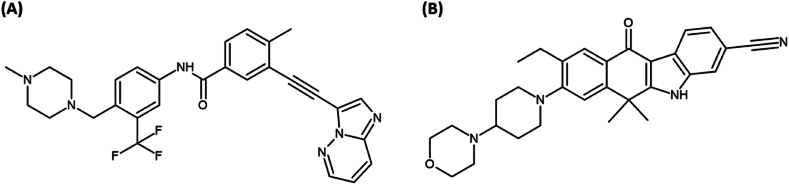
Structures of (A) ponatinib and (B) the internal standard (alectinib).

Ponatinib (purity >99 %; Fig. 1A) and alectinib (purity >99 %; Fig. 1B) were purchased from MedChemExpress (Monmouth Junction, NJ, USA). Dimethyl sulfoxide (DMSO), potassium dihydrogen phosphate, potassium hydrogen phosphate, N,N-dimethylacetamide (DMAC), cremophor EL, and 2-hydroxypropyl-beta-cyclodextrin (HPβCD) were purchased from Sigma-Aldrich (St. Louis, MO, USA). Heparinized blank Sprague Dawley (SD) rat plasma was obtained from Innovative Research, Inc. (Novi, MI, USA). Heparinized rat thoracic jugular vein catheters were purchased from Braintree Science, Inc. (Braintree, MA, USA). HPLC-grade methanol and acetonitrile were obtained from Fisher Scientific (Fair Lawn, NJ, USA) and water was purchased from J.T. Baker (Phillipsburg, PA, USA). All other reagents were of analytical grade and were used without further purification.

### 2.2 Factor screening study

To determine the critical method parameters (CMPs) that significantly affect the critical analytical attributes (CAAs) of ponatinib in rat plasma, a seven-factor, eight-run screening design was used. To develop a sensitive and rapid analytical method, the peak areas and retention times were included as the CAAs. As the analysis was conducted using plasma samples, which contain a variety of endogenous substances, the resolutions of the analyte peak with the preceding peak (resolution 1) and the following peak (resolution 2) were included as CAAs. The experimental factors included the composition of the organic phase, the ratio of organic solvent in the mobile phase, flow rate, buffer pH, buffer strength, injection volume, and column oven temperature. Each factor was tested at two levels, as presented in Table 1, and encoded in −1 (low level) and 1 (high level), respectively, for analysis. Plasma samples containing 10 ng/mL ponatinib were pretreated with methanol and used in the experimental runs. The data obtained from these experiments were subsequently analyzed by fitting them to a linear polynomial model, with the interaction terms deliberately omitted to focus on the main effects. Statistical tools, such as half-normal plots and Pareto charts, were used to quantitatively identify the influence of each factor on CAAs.

**Table 1:** Seven-factor eight-run Taguchi design matrix to screen the method variables.

Run	Method variables						
	Acetonitrile in organic phase (%)	Organic solvent ratio (%)	Flow rate (mL/min)	Buffer pH	Buffer strength (mM)	Injection volume (μL)	Column oven (°C)
1	50	70	1.2	7	15	20	30
2	50	50	0.8	7	5	20	40
3	100	50	1.2	5.8	5	20	30
4	50	50	0.8	5.8	15	10	30
5	50	70	1.2	5.8	15	10	40
6	100	70	0.8	5.8	5	20	40
7	100	50	1.2	7	15	10	40
8	100	70	0.8	7	5	10	30

### 2.3 Method optimization using Box-Behnken design

The CMPs were optimized using a three-factor Box-Behnken design (BBD). Each CMP was tested at three levels of low (−1), middle (0), and high (1) for the following variables: organic solvent 50, 60, and 70 %; flow rate 0.8, 1, and 1.2 mL/min; and buffer 5, 10, and 15 mM. To estimate the experimental error and improve the model precision, 15 experimental runs were performed, including 12 at the midpoints of the edges of the cube and three at the center point. Chromatographic optimization was performed using Design Expert® software version 13.0.5.0 (Stat-Ease Inc., Minneapolis, MN, USA), with a quadratic polynomial model and interaction terms for data analysis. A coefficient analysis was performed using the following polynomial equation for each CAA:

$$Y=\beta_{0}+\beta_{1}A+\beta_{2}B+\beta_{3}C+\beta_{4}\text{AB}+\beta_{5}\text{AC}+\beta_{6}\text{BC}+\beta_{7}A^{2}+\beta_{8}B^{2}+\beta_{9}C^{2}$$

where A (organic solvent ratio), B (flow rate), and C (buffer strength) are the CMPs determined using the factor screening assay, β_0_ is the intercept of the polynomial model, and β_1_ to β_9_ are the coefficients of the model terms.

Response surface methodology was used to determine the effects of CMPs on CAAs and the interactions between these factors. Chromatographic optimization using desirability was applied to balance multiple CMPs, such as maximizing the peak area, minimizing the retention time, and achieving resolutions 1 and 2 greater than 1.5. The final chromatographic conditions were determined via numerical optimization. On the heels of numerical optimization, graphical optimization was also carried out to define the analytical design space and identify the location of the optimized solution.

We conducted model validation by performing ponatinib at five randomly selected conditions within the design space. The observed experimental results were compared with the predicted values from the fitted model, and the % prediction error was calculated using the formula:

$$\%\;prediction\;error\;\left(PE\right)=\left|\frac{observed\;value-predicted\;value}{predicted\;value}\right|\times 100$$

### 2.4 Chromatographic conditions

An Agilent 1200 Series HPLC system (Agilent Technologies, Santa Clara, CA, USA) equipped with a fluorescence detector was used. A reverse-phase HPLC column (Gemini® NX-C18; 250 × 4.6 mm, particle size 5 μm; Phenomenex, Torrance, CA, USA), pre-warmed to 30 °C, was used for the chromatographic separation of ponatinib and the internal standard (IS; alectinib) from endogenous substances. The mobile phase consisted of 15 mM potassium phosphate buffer (pH 5.8) in water and acetonitrile (40:60 v/v) at a flow rate of 0.8 mL/min. The run time was 10.5 min per sample, with the temperature of the autosampler maintained at 10 °C. The excitation and emission wavelengths were set to 327 and 461 nm, respectively. Instrument control and data analysis were performed using the Agilent ChemStation software (Agilent Technologies).

### 2.5 Sample preparation

A 20 μL aliquot of rat plasma was mixed with 100 μL of methanol containing 100 ng/mL of the internal standard (IS; alectinib) to precipitate the plasma proteins. The mixture was then vortexed for 4 min and centrifuged at 12,000 rpm for 4 min at 4 °C. The supernatant was then transferred to an LC vial (Agilent Technologies), and 10 μL was injected into the HPLC system.

### 2.6 Standard and quality control samples

Stock solutions of ponatinib and the IS were prepared at a concentration of 10 mg/mL in DMSO. Subsequently, standard working solutions of ponatinib were prepared by serial dilution of the stock solution with 70 % methanol in water. The IS solution was prepared in methanol to a final concentration of 100 ng/mL. To prepare the standards, 2 μL of the standard working solution was spiked into 18 μL of blank rat plasma to yield final concentrations of 1, 2, 5, 20, 40, 100, 250, 750, and 1000 ng/mL. Quality control (QC) samples were prepared using an identical procedure, resulting in final concentrations of 3 (low QC, LQC), 60 (middle QC, MQC), and 800 ng/mL (high QC, HQC).

### 2.7 Method validation

The analytical method for ponatinib was validated according to the FDA and EMA guidelines for selectivity, sensitivity, linearity, intra-/inter-day accuracy and precision, matrix effect, recovery, carryover effect, and dilution linearity. Selectivity was evaluated using blank plasma samples from six rats. The sensitivity of the analytical method was evaluated by determining the lowest limit of quantification (LLOQ), which demonstrated acceptable accuracy and precision with an analyte response at least five times greater than that of the zero blank. The linearity of the method was determined by analyzing standard samples containing ponatinib concentrations ranging from 1 to 1000 ng/mL, processed as previously described, and analyzed using linear regression on a calibration curve. Accuracy and precision were determined at four different concentrations (LLOQ, LQC, MQC, and HQC) prepared independently from the calibration standards. These evaluations were conducted over three days (n = 5 per day). The accuracy acceptance criteria for the inter- and intra-assay were within ±15 % of the nominal concentrations, except for the LLOQ, where the accuracy was required to be within ±20 %. The precision of the method was determined by calculating the relative standard deviation (RSD) at each concentration level. The acceptance criterion for precision was RSD ≤15 %, except for the LLOQ, whose acceptance criterion was RSD ≤20 %.

Recovery and matrix effects were quantitatively evaluated at three QC levels, as previously described [[23], [24], [25]]. The recovery was determined by dividing the mean peak area of the analyte added before precipitation by that of the analyte spiked into the matrix post-precipitation. The matrix effect was evaluated by comparing the mean peak area of the analyte in the post-precipitation matrix with its peak area when spiked into a neat mobile phase. The recovery and matrix effects were calculated for the IS (100 ng/mL) in the same manner.

The stability of ponatinib in rat plasma was determined under various conditions using LQC and HQC samples in triplicate. The stability tests included bench-top exposure at room temperature for 2 h, long-term storage at −20 °C for 3 weeks, three freeze-thaw cycles, and 24-h autosampler storage at 10 °C. The stability of ponatinib was determined by comparing the measured concentrations with nominal values.

The dilution integrity was evaluated to ascertain the suitability of this analytical method for samples with concentrations above the upper limit of quantification (ULOQ). Rat plasma samples were prepared at a concentration ten times higher than the HQC level and diluted 10-fold with blank plasma. Thereafter, the samples were processed using the aforementioned procedure. To assess the potential for carryover, blank samples were analyzed immediately after the ULOQ samples. The carryover potential was determined by comparing the peak areas of blank samples with those of LLOQ samples and considered acceptable when the peak area of the blank sample was less than 20 % of that of the LLOQ samples.

### 2.8 In vivo pharmacokinetic study of ponatinib

Animal experiments were conducted following the guidelines of the Korea Research Institute of Bioscience and Biotechnology (KRIBB-AEC-20309). Male SD rats (Koatech Inc.; Pyeongtaek, Gyeonggi, Republic of Korea) were acclimated to laboratory conditions for at least one week before experimentation. Animals were permitted *ad libitum* access to food and water, except during the 8 h fasting period before drug administration; however, water was continuously available.

Rats were administered ponatinib, prepared in a DMAC, cremophor EL, and 20 % HPβCD) in water (10:10:80) for intravenous (IV; 4 mg/kg) and oral administration (7.5 mg/kg). Blood samples (approximately 100 μL) were collected in lithium heparin-treated tubes through the jugular vein at 5, 15, and 30 min, and 1, 3, 6, 12, 24, 36, and 48 h for the IV group (n = 3) and at 30 min and 1, 2, 4, 6, 8, 12, 24, 36, and 48 h post-dose for the oral group (n = 3). The samples were centrifuged at 13,500×*g* for 10 min at 4 °C. Plasma was stored at −20 °C until analysis.

Plasma ponatinib concentrations were determined using the HPLC-FLD method developed in this study. Pharmacokinetic parameters were calculated using non-compartmental analysis via WinNonlin software (Pharsight Corporation, Mountain View, CA, USA). The key parameters were the area under the plasma concentration-time curve from zero to the last quantifiable time point (AUC_last_) and infinity (AUC_inf_), both of which were calculated using the linear trapezoidal method. The terminal half-life (T_1/2_) was derived from the elimination constant at the terminal phase (λ), and total clearance (CL) and the steady-state volume of distribution (V_ss_) were calculated. The maximum plasma concentration (C_max_) and time to reach C_max_ (T_max_) were obtained directly from the plasma concentration-time graph. The oral bioavailability (F) was determined by dividing the dose-normalized AUC_inf_ value following oral administration by that following IV administration.

## 3 Results

### 3.1 Optimization of the chromatographic conditions

The main effects of the factors were determined using a factorial design based on the Taguchi orthogonal array design, which allowed the assessment of the individual impacts of each factor without considering their interaction effects. Analysis of variance (ANOVA) revealed that the selected factorial model for each response was statistically significant, with coefficient of determination greater than 0.9, indicating a strong fit of the models to the data. Further analysis using Pareto ranking revealed the significant contributions of each factor to the various responses. For retention time, the most influential factors were the organic solvent ratio in the mobile phase, flow rate, and buffer pH. In the case of peak area, the organic solvent ratio in the mobile phase, flow rate, and buffer strength were identified as the key factors. For resolution 1, buffer strength, buffer pH, and column temperature played the most significant roles, while for resolution 2, the organic solvent ratio in the mobile phase, flow rate, and buffer strength were the most important factors affecting the response. Considering the overall significance of these results, the organic solvent ratio in the mobile phase, flow rate, and buffer strength were selected as the CMPs for further optimization studies. These factors were chosen because of their statistically significant influence on multiple CAAs, ensuring that further optimization would focus on the parameters most likely to improve the performance of the chromatographic method.

In this study, BBD was employed to determine the optimal chromatographic conditions to maximize the detection response, minimize the running time, and achieve complete separation from other substances in the plasma. Thus, the responses included peak area, retention time, resolution 1, and resolution 2. The quadratic polynomial models for each response variable and the statistical parameters derived from ANOVA are presented in Table 2. All response variable models were statistically significant (p < 0.001) and the R^2^ was above 0.95, indicating a high degree of fit. No significant lack of fit was observed in any of the models (p > 0.05). In all test runs, the prediction errors were less than 5 %, confirming the accuracy, robustness, and reliability of the fitted model across the design space (Table S1).

**Table 2:** Coefficients of polynomial equations for the quadratic model and their statistical parameters.

Coefficient code	Polynomial coefficients for response variables			
	Peak area (A)	Retention time (B)	Resolution 1 (C)	Resolution 2 (D)
β_0_	24.72	4.97	7.51	2.14
β_1_	1.63	−1.96	−0.52	−2.05
β_2_	−6.59	−1.26	0.03	−0.46
β_3_	−0.54	−0.71	−1.10	0.13
β_4_	0.57	0.38	0.02	−0.28
β_5_	3.67	0.45	−1.21	−0.70
β_6_	0.91	0.15	0.48	−0.20
β_7_	0.08	0.94	−2.01	2.70
β_8_	4.31	0.27	−4.55	1.18
β_9_	2.43	0.53	0.22	−1.07
Adjusted R^2^	0.9618	0.9989	0.9618	0.9972
*p value*	0.0004	<0.00001	0.0004	<0.00001

**Fig. 2:**
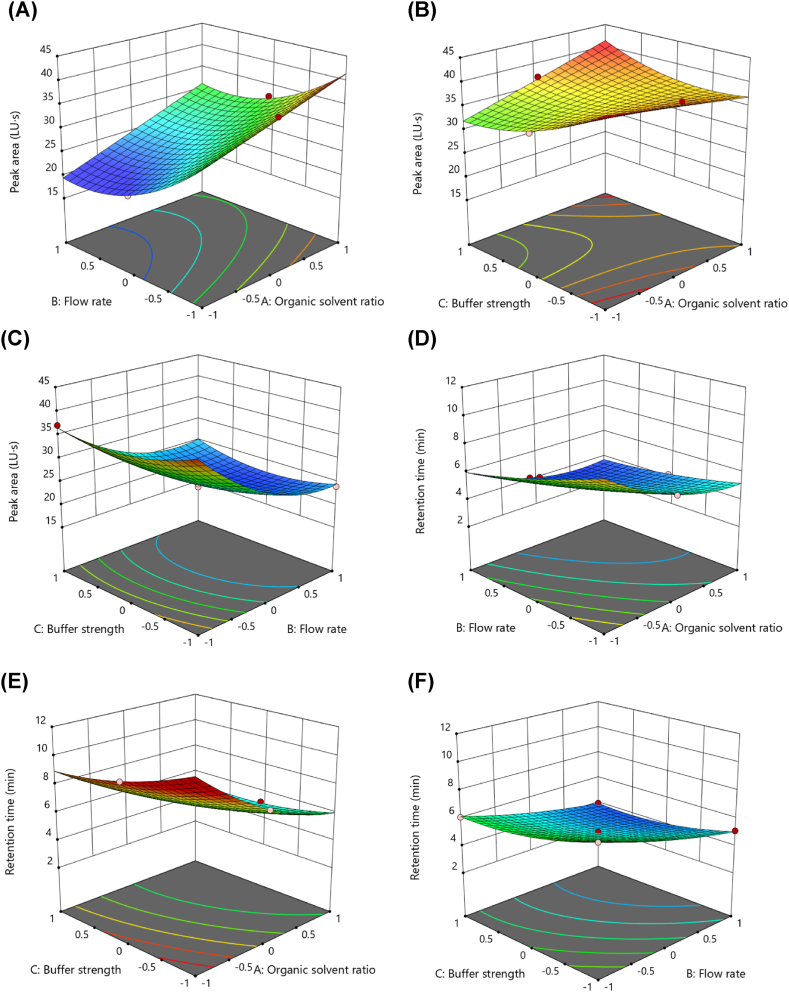

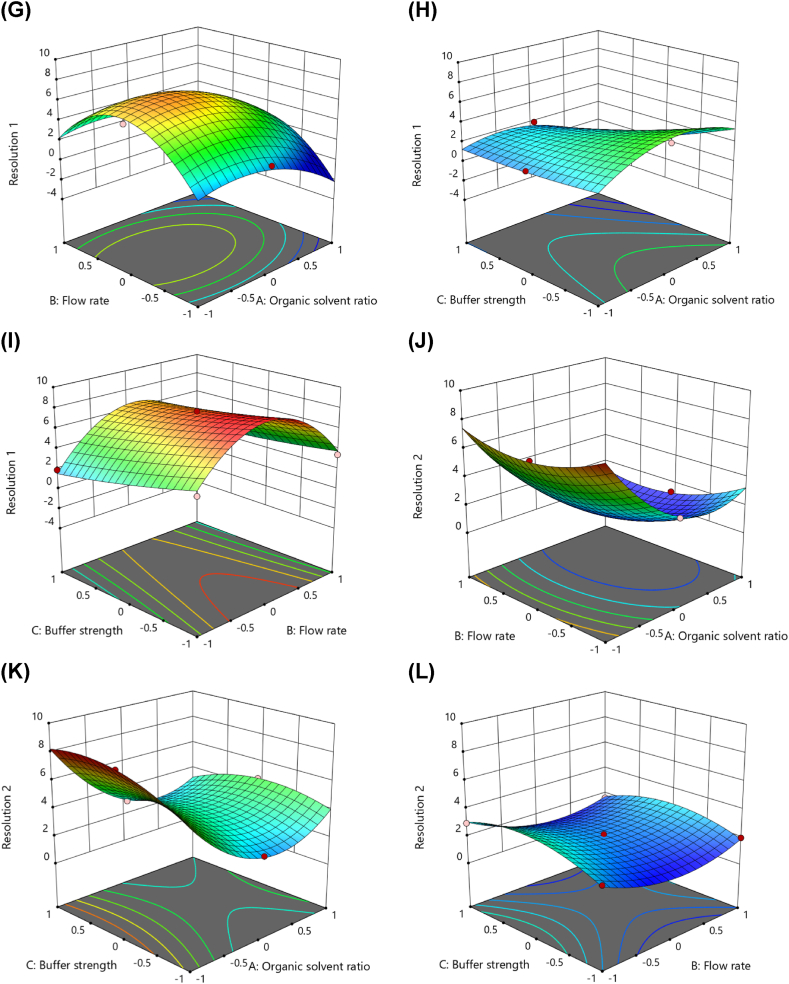
3D-response surface plots showing the influence of CMPs on CAAs. Effect of organic solvent ratio, flow rate, and buffer strength on the peak area (A–C), retention time (D–F), resolution 1 (G–I), and resolution 2 (J–L). Each CMP was tested with low (−1), middle (0), and high (1) levels of the following variables: organic solvent 50, 60, and 70 %; and flow rate 0.8, 1, and 1.2 mL/min; and buffer 5, 10, and 15 mM.

A curvilinear response surface was observed for the effects of CMPs on the peak area (Fig. 2 and Fig. S1). An increase in the flow rate was found to result in a reduction in the peak area, irrespective of the level of organic phase ratio and buffer strength. As the organic phase ratio increased, the peak area tended to increase at higher buffer strengths and slightly decrease at lower buffer strengths. In terms of buffer strength, different patterns were observed in the peak area depending on the organic solvent ratio. At a lower organic solvent ratio, a negative correlation was observed between peak area and buffer strength; however, at a higher organic solvent ratio, a positive correlation was found between peak area and buffer strength from the midpoint onward.

The organic solvent ratio and flow rate negatively affected the retention time. In contrast, a negative correlation was observed between buffer strength and retention time at a low organic solvent ratio; however, minimal effects on retention time with a U-shaped curve were observed at high organic solvent ratios.

The response surface of the organic solvent ratio and flow rate exhibited a "dome-like" shape on resolution 1. In terms of buffer strength, a clear negative correlation was observed with resolution 1 at all flow rate levels and higher organic solvent ratios. Conversely, at lower organic phase ratios, a minimal increase in resolution 1 occurred with an increase in buffer strength. A "U-shaped" response surface was observed in the relationship between the organic solvent ratio and flow rate with respect to resolution 2. In contrast, resolution 2 tended to increase in the center of the buffer strength range and decrease toward both ends, which represents a "saddle shape" type response surface.

Based on numerical optimization, the optimal chromatographic conditions for the analysis of ponatinib in rat plasma were 60 % organic solvent ratio in the mobile phase (0), flow rate of 0.8 mL/min (−1), and buffer strength of 15 mM (1), which achieved a desirability score of 0.820, and the optimal solution was demarcated in the design space overlay plot (Fig. S2).

### 3.2 Selectivity and sensitivity

**Fig. 3:**
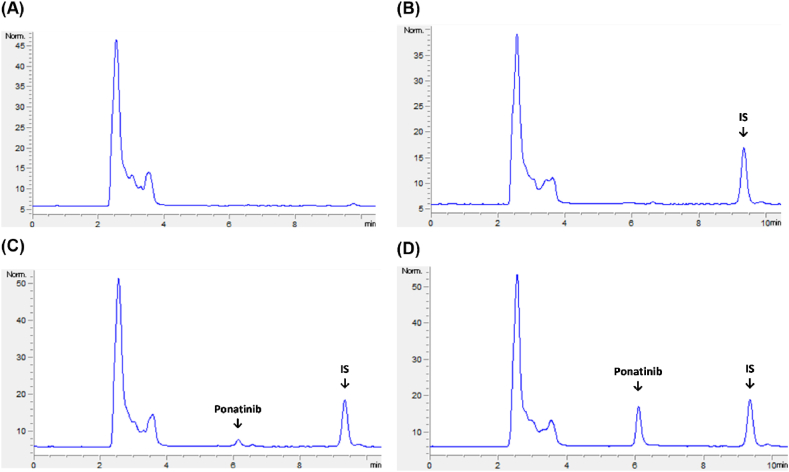
Representative chromatograms of ponatinib and the internal standard (IS; alectinib). (A) Blank rat plasma; (B) blank rat plasma spiked with the IS (100 ng/mL); (C) LLOQ (1 ng/mL) sample in rat plasma; (D) rat plasma sample collected at 1 h after oral administration of ponatinib at a dose of 7.5 mg/kg to rats.

Chromatograms (Fig. 3) of blank plasma, zero blank plasma spiked with the internal standard (IS), plasma at the LLOQ (1 ng/mL), and plasma samples from rats 1 h after oral administration of ponatinib (7 mg/kg) exhibited distinct separation without significant interference at retention times of 6.1 min for ponatinib and 9.3 min for IS. The LLOQ of 1 ng/mL, determined following regulatory guidelines, was validated with sufficient accuracy and precision across six replicates in three runs, confirming the selectivity and sensitivity of the method for detecting ponatinib in rat plasma (Table 3).

**Table 3:** Accuracy and precision of ponatinib quantification in rat plasma.

QC	Nominal conc. (ng/mL)	Inter-day (n = 15)			Intra-day (n = 5)		
		Measured conc. (ng/mL; mean ± SD)	Accuracy (%; mean ± SD)	Precision (RSD, %)	Measured conc. (ng/mL; mean ± SD)	Accuracy (%; mean ± SD)	Precision (RSD, %)
LLOQ	1	1.02 ± 0.08	102 ± 8	7.64	1.02 ± 0.08	102 ± 8	7.75
LQC	3	2.94 ± 0.22	98.1 ± 7.2	1.08	3.00 ± 0.28	100 ± 9	9.19
MQC	60	58.7 ± 3.5	97.9 ± 5.7	1.13	58.2 ± 1.5	97.0 ± 2.5	2.57
HQC	800	823 ± 52	103 ± 6	0.99	792 ± 13	99.0 ± 1.6	1.65

Accuracy, calculated as measured concentration/nominal concentration × 100 %; RSD, relative standard deviation, calculated as the standard deviation of concentration/mean concentration × 100 %; SD, standard deviation.

**Table 4:** Recovery and matrix effect of ponatinib and the internal standard in rat plasma (mean ± SD, n = 5).

	Ponatinib			Internal standard (alectinib)
	LQC (3 ng/mL)	MQC (60 ng/mL)	HQC (800 ng/mL)	100 ng/mL
Recovery (%)^a^	92.2 ± 12	107 ± 3	101 ± 7	101 ± 12
Matrix effect (%)^b^	87.7 ± 10.7	87.3 ± 2.7	83.3 ± 3.6	107 ± 4

a Matrix effect (%) = mean peak area of an analyte added post-precipitation/mean peak area of an analyte in a neat analyte solution × 100.

b Recovery (%) = mean analyte peak area of an analyte added before precipitation/mean peak area of an analyte added after precipitation × 100.

**Table 5:** Stability of ponatinib in rat plasma (mean ± SD, n = 3).

Storage condition	Stability (%, mean ± SD)	
	LQC (3 ng/mL)	HQC (800 ng/mL)
Bench top (room temperature for 2 h)	108 ± 6	91.5 ± 2.1
Long term (−20 °C for 3 weeks)	93.1 ± 7.9	96.4 ± 4.3
Freeze-thaw (3 cycles)	89.1 ± 7.0	92.1 ± 5.0
Autosampler (10 °C for 24 h)	93.4 ± 1.3	95.2 ± 11.1

The stability (%) was calculated by dividing the measured concentration after the designated storage condition by the nominal condition.

**Table 6:** Pharmacokinetic parameters of ponatinib after intravenous or oral administration to rats (mean ± SD, n = 3).

Parameter	IV (4 mg/kg)	PO (7.5 mg/kg)
T_max_ (h)	–	5.34 ± 2.31
C_max_ (ng/mL)	–	308 ± 11
T_1/2_ (h)	6.84 ± 0.22	8.75 ± 0.67
AUC_last_ (ng·h/mL)	6100 ± 730	5400 ± 270
AUC_inf_ (ng·h/mL)	6140 ± 750	5520 ± 280
CL (mL/h/kg)	657 ± 75	–
V_ss_ (mL/kg)	5390 ± 200	–
F (%)^a^	–	48.2 ± 3.9

AUC, area under the plasma concentration-time curve; CL, total clearance; C_max_, maximum plasma concentration; F, bioavailability; SD, standard deviation; T_1/2_, terminal half-life; T_max_, time to reach C_max_; V_ss_, steady-state volume of distribution.

a Calculated using the dose-normalized AUC_PO_/dose-normalized AUC_IV_ × 100.

### 3.3 Linearity

The linearity of the method was confirmed over the concentration range of 1–1000 ng/mL in rat plasma, with a calibration curve described by the regression equation: *y = 2.88 x – 0.018,* where *x* represents the concentration ratio of ponatinib/IS and *y* denotes the peak area ratio of ponatinib/IS. The coefficient of determination consistently exceeded 0.99 in each validation run, confirming the linearity of the method. Furthermore, in each validation run, more than 75 % of the standard samples satisfied the acceptance criteria, which specify that calculated concentrations must be within ±15 % of the nominal concentration, except for the LLOQ, which should be within ±20 % of the nominal concentration, as per the guidelines.

### 3.4 Method validation

The analytical method was validated via comprehensive assessments of accuracy and precision using the LLOQ, LQC, MQC, and HQC samples (1, 3, 60, and 800 ng/mL). The inter-day accuracy ranged from 97.9 % to 103 %, with an RSD of less than 10 %, whereas the intra-day accuracy ranged from 97.0 % to 102 %, with an RSD value of less than 10 %. These results highlight the reliability of this method for accurate and precise quantification of ponatinib in rat plasma.

Recovery and matrix effects were evaluated at three QC levels for ponatinib (3, 60, and 800 ng/mL) and one concentration for the IS (100 ng/mL). The mean recovery for ponatinib ranged from 92.2 % to 107 %, whereas that for IS was 101 %. This result indicates that both the analyte and IS could be adequately recovered using the sample processing method. The mean peak area ratios in the post-precipitation matrix to the ones in the neat solution were found to exhibit consistency across the different QC levels, with values ranging from 83.3 % to 87.7 % (Table 4).

Stability of ponatinib was evaluated at two QC levels (LQC and HQC) under various conditions (Table 5). Samples stored at room temperature for 2 h showed stabilities ranging from 91.5 % to 108 %, whereas those stored at −20 °C for 3 weeks exhibited stabilities between 93.1 % and 96.4 %. Stability in post-preparative samples (storage at 10 °C for 10 h, identical to the autosampler conditions) and after repeated freeze-thaw cycles ranged from 89.1 % to 92.1 % and 93.4 %–95.2 %, respectively, with RSD values within acceptable ranges. These results indicate that ponatinib was stable under all tested conditions.

Dilution integrity was validated by diluting the samples prepared ten times higher than the HQC level by ten-fold. This dilution resulted in an accuracy of 89.1 % and an RSD of 5.7 %. No carry-over effect was observed in the blank sample analyzed immediately following the ULOQ samples. When blank samples were analyzed immediately after the ULOQ samples, no carryover effects was observed.

### 3.5 Pharmacokinetic study of ponatinib in rats

The analytical method developed for ponatinib was applied to a pharmacokinetic study in rats. Ponatinib was administered intravenously or orally at doses of 4 or 7.5 mg/kg, respectively, and plasma samples were collected at designated time points to measure ponatinib concentration. In the analytical run, ponatinib in rat plasma was quantified according to the FDA and EMA guidelines, which require that at least 67 % of the QC samples and at least 50 % of the QCs per level be within ±15 % of the nominal concentrations. Samples exceeding the ULOQ were diluted ten-fold to be within the linear range of the calibration curve.

**Fig. 4:**
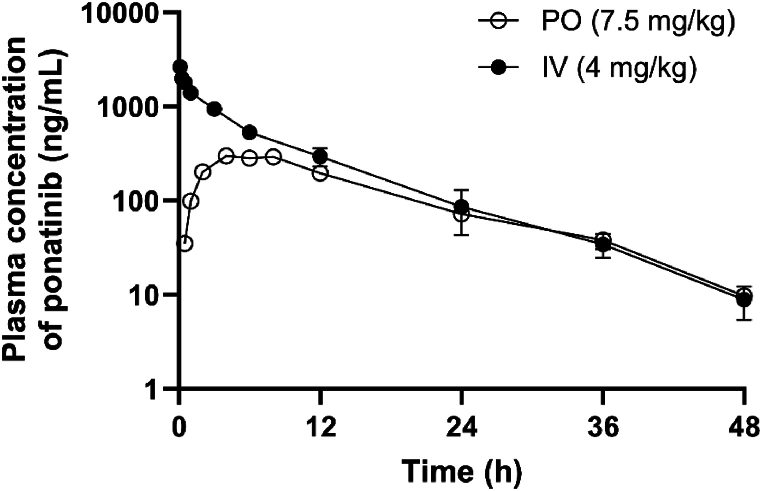
Plasm concentration-time curve of ponatinib after intravenous (IV, 4 mg/kg; closed circles) or oral administration (PO, 7.5 mg/kg; open circles) to rats. Each data point represents mean ± SD (n = 3 for each group).

After IV administration of ponatinib of 4 mg/kg, the plasma concentration continuously decreased, with a terminal T_1/2_ of 6.84 ± 0.22 h. The AUC_inf_ and V_ss_ were 6140 ± 750 ng h/mL and 5390 ± 200 mL/kg, respectively, and the total clearance was 657 ± 75 mL/h/kg. Following oral administration of ponatinib at 7.5 mg/kg, plasma concentration increased, with T_max_ of 5.34 ± 2.31 h and C_max_ of 308 ± 11 ng/mL; thereafter, the concentrations gradually decreased. Assuming an absolute bioavailability (F) of 1 for IV administration, the oral bioavailability of ponatinib in rats was 48.2 % (Table 6 and Fig. 4).

## 4 Discussion

The objective of this study was to develop and validate a sensitive and selective bioanalytical method for quantifying ponatinib in rat plasma using HPLC-FLD. By systematically analyzing the variables that influence the analysis and optimization of the analytical conditions using the AQbD method, we developed a method with low variability and high reliability. The LLOQ was 1 ng/mL, with a quantification range of 1–1000 ng/mL, ensuring a wide range of detectable concentrations. This method was free of carryover effects and demonstrated consistent recovery and matrix effects. The developed assay was successfully applied to a pharmacokinetic study in rats. As various preclinical studies in animals, such as rodents, will precede the expansion of the indications for ponatinib, the results of this study will help extend its applications. Furthermore, the analytical method developed in this study for rat plasma can be used to analyze human plasma with slight modifications.

In previous studies, analytical methods for ponatinib were developed using HPLC-UV and HPLC-MS/MS; however, our method offers clear advantages. Yasu et al. (2018) used HPLC-UV to quantify ponatinib in the range of 5–250 ng/mL. The run time of this method was 15 min and 400 μL of plasma and complex solid-phase extraction (SPE) were required for sample preparation [26]. The requirement for complex procedures, such as SPE, often leads to longer processing times and higher operational costs, limiting the practicality of the method for routine analysis. Abumiya et al. (2018) reduced the LLOQ to 1 ng/mL using the HPLC-UV method; however, a 30-min run time and similarly complex sample preparation were required [27]. A long runtime may not be feasible for high-throughput analyses, particularly when handling a large number of samples. The high LLOQ and long run times in the HPLC-UV method might be due to co-existing endogenous substances in the plasma that absorb light at similar wavelengths, ultimately interfering with the accurate quantification of ponatinib. The analytical method for the determination of ponatinib using LC-MS/MS has been reported; however, this method required 50 μL of plasma and complex liquid-liquid extraction (LLE) for sample preparation [20]. Although LC-MS/MS provides higher sensitivity and specificity than HPLC-UV, the complexity and cost of instrumentation, and labor-intensive sample preparation can act as limiting factors. Furthermore, LLE involves multiple steps and solvent usage, which can generate variability and reduce the throughput. The analytical method developed in this study, which uses a fluorescence detector, offers significant improvements over traditional methods. Fluorescence detection is inherently more sensitive than UV detection and enabled the quantification of ponatinib in the range of 1–1000 ng/mL using 20 μL of plasma samples and a shorter run time in this study. Simple protein precipitation for sample preparation streamlines the process, reducing the risks of error and variability associated with more complex preparation techniques. Accordingly, our method is more efficient and faster for plasma analysis than prior methods, demonstrating its superior performance compared with these previous methods.

In this study, we used a systematic approach combining the Taguchi method's seven-factorial 8 run design and the BBD to optimize the analytical conditions for quantifying ponatinib in biological samples. Initially, the Taguchi method was employed to screen and identify critical factors affecting the analytical method. Plasma, as a biological matrix, presents significant challenges due to the presence of various endogenous substances that can interfere with analytical measurements. The Taguchi method is particularly effective in addressing this variability by allowing to systematically identify and optimize the conditions that minimize interference and enhance the accuracy of the analysis. Additionally, the Taguchi method offers a high level of efficiency in experimental design. By employing orthogonal arrays, we were able to investigate multiple factors simultaneously with a reduced number of experiments (i.e., eight runs). As a result, the organic solvent ratio in the mobile phase, flow rate, and buffer strength were found to significantly influence the CAAs. After factor screening, BBD was used to fine-tune and optimize the analytical conditions. BBD, a type of response surface methodology, was chosen because it is particularly well-suited for developing and optimizing quantitative analytical methods in complex matrices like plasma. The plasma matrix can introduce variability that affects the interaction between factors, such as solvent composition and flow rate, making it crucial to accurately model these interactions. BBD effectively fits second-order (quadratic) models and explores these interactions, allowing us to pinpoint optimal conditions that account for the matrix's complexity. Compared with the full factorial design, which requires an extremely large number of experiments, BBD required only 15 runs to effectively develop the model response surface. This design ensured the generation of a robust and predictive model by accurately estimating the main effects and interactions among the factors. The combination of these two methodologies enabled systematic and economic optimization of the analytical procedure. This hybrid approach not only enhances the reliability and robustness of the analytical method but also aligns with best practices in method development by incorporating comprehensive statistical techniques to ensure high-quality results.

In this study, the response surface analysis revealed complex interactions between the CMPs and the CAAs, highlighting the significant roles of organic solvent ratio, flow rate, and buffer strength. The inverse relationship between flow rate and peak area suggests that slower flow rates are key to maximizing detection sensitivity, while the varying effects of buffer strength and organic solvent ratio on peak area emphasize the need for balance in optimizing method performance. The "dome-like" response for resolution 1 indicates a non-linear interaction between flow rate and organic solvent ratio, suggesting that optimal separation occurs within a narrow range of conditions. Similarly, the saddle-shaped response for resolution 2 shows that buffer strength is crucial for maintaining separation quality, as resolution improves at mid-range buffer strengths but diminishes at the extremes. These findings underscore the importance of fine-tuning CMPs within the design space to achieve consistent and reliable chromatographic performance.

In AQbD-driven analytical method optimization, typical responses include the theoretical plate number, tailing factor, resolution, peak area, and runtime. However, appropriate responses must be selected based on the characteristics of the samples and analytical instruments used. In this study, we used HPLC-FLD, which may have drawbacks, such as long run times and potential difficulties in separating interference. Furthermore, due to the need to detect low drug concentrations in biological matrices, achieving sufficient sensitivity is of utmost importance. To address these challenges, we set our response variables to minimize the run time, maximize the peak area, and achieve a resolution of at least 1.5 between adjacent peaks. The final selected method revealed no influence from co-existing interferences, achieved detection limits as low as 1 ng/mL, and had a shorter run time than conventional HPLC-UV methods. In this study, an analytical method that considers the key points in drug quantification in plasma was successfully developed, highlighting the importance of tailored response settings for AQbD-driven optimization.

Using the validated analytical method, we calculated the PK parameters by analyzing plasma samples obtained after administering ponatinib to rats via IV or PO route. Our results showed a difference of less than 1.5-fold when compared to the data from the FDA's clinical pharmacology and biopharmaceutics review [28]. However, a difference of approximately 3-fold was observed compared to the data reported by Wang et al. [20]. The observed differences may be due to variations in the vehicles used, which can influence drug absorption and kinetics or any other experimental conditions. Further research is needed to explore these differences.

This study has certain limitations that should be acknowledged. First, while we successfully identified CMPs through experimental data and optimization processes, a formal risk assessment, such as Failure Mode and Effect Analysis (FMEA), was not conducted. A structured risk assessment could have provided additional insights into potential sources of variability and risks in the method. Future studies incorporating this step could further enhance the robustness and reliability of the analytical method. Additionally, while we achieved a shorter run time (10.5 min) compared to the UV method (15 min), additional efforts, such as exploring the use of a shorter column with smaller particle sizes or other advanced techniques, should be considered to further improve method efficiency without compromising sensitivity or resolution.

## 5 Conclusion

In this study, we developed and validated a highly sensitive and selective HPLC-FLD method to quantify ponatinib in rat plasma. The application of the AQbD approach, which incorporates both the Taguchi and BBD methods, enabled efficient optimization of the analytical parameters, resulting in a method that is both robust and reliable. This newly developed method required a minimal sample volume and a shorter run time, providing superior sensitivity and streamlined sample preparation compared to conventional HPLC-UV and LC-MS/MS methodologies. Consequently, this method is highly suitable for routine analytical applications and supports further pharmacokinetic investigations in preclinical and clinical settings.

## Ethics statements

Animal experiments were conducted following the guidelines of the Korea Research Institute of Bioscience and Biotechnology (KRIBB-AEC-20309).

## CRediT authorship contribution statement

**Nahyun Koo:** Writing – original draft, Investigation, Formal analysis, Data curation. **Eun Ji Lee:** Writing – original draft, Methodology, Investigation, Formal analysis. **Min Ju Kim:** Writing – original draft, Investigation, Formal analysis, Data curation. **Minjung Park:** Writing – original draft, Investigation, Formal analysis, Data curation. **Kyeong-Ryoon Lee:** Writing – review & editing, Visualization, Formal analysis, Conceptualization. **Yoon-Jee Chae:** Writing – review & editing, Visualization, Supervision, Methodology, Funding acquisition, Conceptualization.

## Declaration of competing interest

The authors declare that they have no known competing financial interests or personal relationships that could have appeared to influence the work reported in this paper.
