# Supplementary material for: Quality by design-based approach for the development of an analytical method for quantifying ponatinib in rat plasma
Source: Heliyon. 2024 Sep 28;10(19):e38637. doi: 10.1016/j.heliyon.2024.e38637 (PMC11471595; doi:10.1016/j.heliyon.2024.e38637)
Supplement: Multimedia component 1 [file mmc1.pdf]

Supplementary Materials

Quality by design-based approach for the development of an analytical method for quantifying ponatinib in rat plasma

Nahyun Koo[1,†], Eun Ji Lee[1,†], Min Ju Kim[2], Minjung Park[2,3], Kyeong-Ryoon Lee[2,3], and Yoon-Jee Chae[1,4,*]

[1] College of Pharmacy, Woosuk University, Wanju 55338, Republic of Korea

[2] Laboratory Animal Resource Center, Korea Research Institute of Bioscience and Biotechnology, Cheongju 28116, Republic of Korea

[3] Department of Biotechnology, University of Science and Technology, Daejeon 34113, Republic of Korea

[4] Research Institute of Pharmaceutical Sciences, Woosuk University, Wanju 55338, Republic of Korea

[†] These authors contributed equally to this work.

[*] **Corresponding author:**

Yoon-Jee Chae

College of Pharmacy and Research Institute of Pharmaceutical Sciences, Woosuk University, Wanju 55338, Republic of Korea

Tel: +82-63-290-1424

E-mail: yjchae@woosuk.ac.kr

Figure Legends

Figure S1. 2D-response surface plots showing the influence of CMPs on CAAs. Effect of organic solvent ratio, flow rate, and buffer strength on the peak area (A−C), retention time (D−F), resolution 1 (G-I), and resolution 2 (J-L). Each CMP was tested with low (-1), middle (0), and high (1) levels of the following variables: organic solvent 50, 60, and 70%; and flow rate 0.8, 1, and 1.2 mL/min; and buffer 5, 10, and 15 mM.

Figure S2. Overlay plot representing the optimized HPLC conditions within the design space (yellow region)

Figure S1.

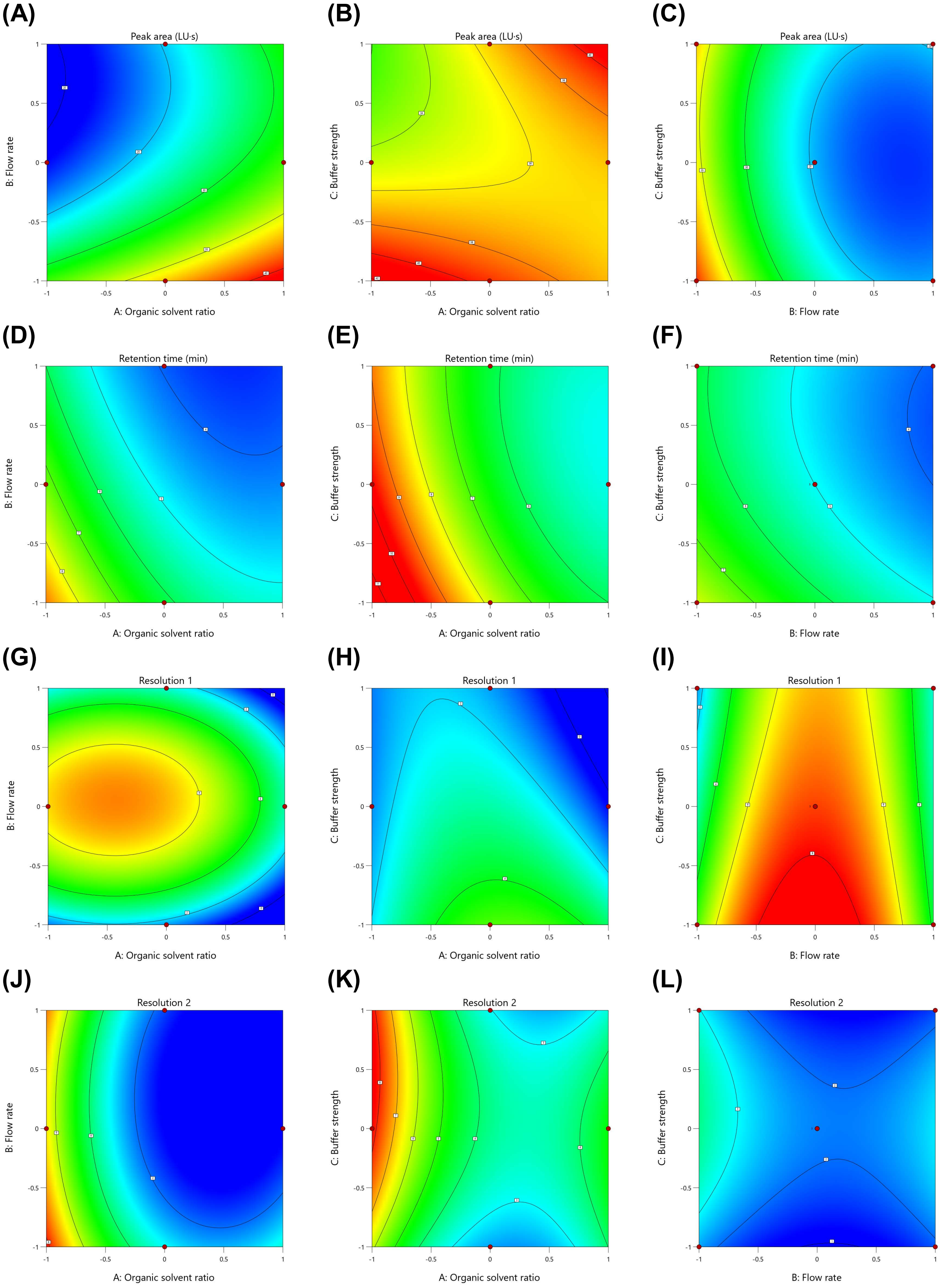
(A)
Peak area (LU·s)
B: Flow rate
A: Organic solvent ratio
(B)
Peak area (LU·s)
C: Buffer strength
A: Organic solvent ratio
(C)
Peak area (LU·s)
C: Buffer strength
B: Flow rate
(D)
Retention time (min)
B: Flow rate
A: Organic solvent ratio
(E)
Retention time (min)
C: Buffer strength
A: Organic solvent ratio
(F)
Retention time (min)
C: Buffer strength
B: Flow rate
(G)
Resolution 1
B: Flow rate
A: Organic solvent ratio
(H)
Resolution 1
C: Buffer strength
A: Organic solvent ratio
(I)
Resolution 1
C: Buffer strength
B: Flow rate
(J)
Resolution 2
B: Flow rate
A: Organic solvent ratio
(K)
Resolution 2
C: Buffer strength
A: Organic solvent ratio
(L)
Resolution 2
C: Buffer strength
B: Flow rate

Figure S2.

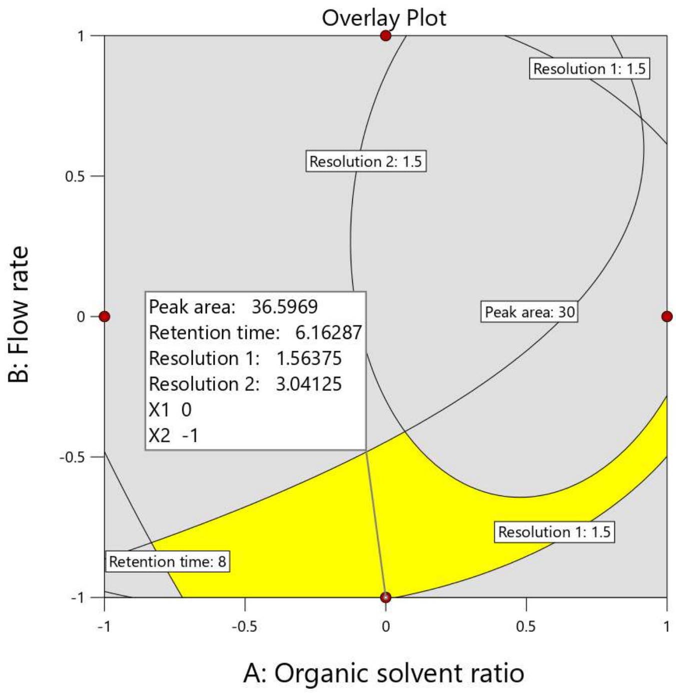

Overlay Plot
Resolution 1: 1.5
Resolution 2: 1.5
Peak area: 36.5969
Retention time: 6.16287
Resolution 1: 1.56375
Resolution 2: 3.04125
X1 0
X2 -1
Peak area: 30
Resolution 1: 1.5
Retention time: 8
1
0.5
0
-0.5
-1
-1
-0.5
0
0.5
1
B: Flow rate
A: Organic solvent ratio

Table S1. Model validation results using the HPLC conditions within design space

No.	Experimental condition	Response (LU·s)	Peak area	Retention time (min)	Resolution 1	Resolution 2
1	Mobile phase: 68%<br>Flow rate: 0.9 mL/min<br>Buffer strength: 12.5 mM	Predicted	31.80	4.50	3.50	2.40
		Observed	30.90	4.61	3.51	2.45
		% PE	2.83	2.44	0.29	2.08
2	Mobile phase: 63%<br>Flow rate: 0.86 mL/min<br>Buffer strength: 12.5 mM	Predicted	32.39	5.21	4.07	2.49
		Observed	33.40	5.18	3.97	2.48
		% PE	3.12	0.58	2.46	0.40
3	Mobile phase: 60%<br>Flow rate: 0.84 mL/min<br>Buffer strength: 11 mM	Predicted	33.20	5.50	3.90	2.90
		Observed	33.00	5.39	3.81	2.99
		% PE	0.60	2.00	2.31	3.10
4	Mobile phase: 55%<br>Flow rate: 0.92 mL/min<br>Buffer strength: 6 mM	Predicted	31.10	7.90	7.20	3.00
		Observed	32.10	7.84	7.02	2.98
		% PE	3.22	0.76	2.50	0.67
5	Mobile phase: 67%<br>Flow rate: 0.86 mL/min<br>Buffer strength: 13 mM	Predicted	33.80	4.80	2.70	2.50
		Observed	34.20	4.79	2.65	2.58
		% PE	1.18	0.21	1.85	3.20
